# Digital Learning Competence and Learning Performance Among Chinese Higher Vocational College Students: A Dual-Path Moderated Mediation Model

**DOI:** 10.3390/bs16060952

**Published:** 2026-06-09

**Authors:** Rongxia Zhuang, Li Liao, Yunbo Liu, Xiaoxi Lin

**Affiliations:** 1 Faculty of Education, Beijing Normal University, Beijing 100875, China; zhuangrx@bnu.edu.cn (R.Z.); 18774882682@163.com (L.L.); 2 Education Bureau of Ganjingzi District, Dalian 116031, China; lynnxiaoxi5210@163.com

**Keywords:** digital learning competence, higher vocational education, learning participation, learning performance, teacher–student interaction, curriculum practicality, moderated mediation

## Abstract

Digital transformation is reshaping technical and vocational education and training (TVET), yet the behavioral processes through which students’ digital learning competence is associated with learning performance remain underexplored. Drawing on Biggs’ presage–process–product (3P) model, this cross-sectional study examined a dual-path moderated mediation model in which active and rule-based learning participation served as differentiated process pathways, while teacher–student interaction and curriculum practicality were specified as contextual moderators. Survey data were collected from 3693 students in Chinese higher vocational colleges. Hierarchical regression and bootstrapped moderated mediation analyses indicated that digital learning competence was positively associated with learning performance. Active learning participation mediated this association, whereas rule-based learning participation did not function as a stable positive mediator. At higher levels of teacher–student interaction and curriculum practicality, digital learning competence showed stronger associations with active learning participation and stronger indirect associations with learning performance. The rule-based pathway appeared more conditional and reflected an externally regulated, prescribed-task-oriented form of behavioral participation, rather than a stable process pathway associated with deep learning. These findings extend the 3P model to digital learning in higher vocational education, differentiate behavioral forms of participation, and highlight the importance of interactive and practice-oriented instructional contexts.

## 1. Introduction

Digital transformation is reshaping technical and vocational education and training (TVET) by changing workplace skill demands and the learning environments in which students develop these skills. Digital skills have become increasingly important for work adaptation, career development, and the quality of vocational training (International Labour Office, 2021; OECD, 2024). In China, where higher vocational education is a major channel for cultivating technical talent, digital learning has become increasingly embedded in teaching practice (Ministry of Education of the People’s Republic of China, 2022; OECD, 2023). However, learning performance is not improved simply because students can operate digital tools. A central behavioral question is how students deploy digital learning competence in learning processes, and under which instructional conditions this competence becomes performance-relevant.

Previous research suggests that digital competence can support learning by strengthening information literacy, knowledge visualization, and online learning processes (Gu & Hu, 2018; Yi, 2023). Yet many studies have focused on whether digital competence matters rather than how it matters. Learning participation is often treated as a single broad construct, although students in higher vocational education may participate in qualitatively different ways. Active participation involves exploration, reflection, and self-directed engagement, whereas rule-based participation emphasizes compliance with instructional norms and task requirements. Studies of vocational students have shown that participation is shaped by institutional and cultural contexts (Y. S. Wang & Wang, 2017; Zhou, 2020; Ye & Nylander, 2024), but limited research has examined whether these distinct participation patterns play different roles in linking digital learning competence to learning performance.

The instructional context surrounding this process also requires further attention. In higher vocational education, teacher–student interaction and curriculum practicality are especially important because learning is closely tied to feedback, task performance, and applied practice. Prior studies have shown that teacher–student interaction is associated with students’ learning gains (Ding & Yang, 2022), while curriculum practicality may shape whether students can apply digital capabilities in meaningful learning situations (Deng et al., 2023). Nevertheless, current research has not systematically explained how these contextual factors may condition the pathways through which digital learning competence relates to learning performance via different forms of participation.

These issues are particularly important in the Chinese higher vocational context. China has the world’s largest vocational education system, and the effectiveness of higher vocational education is closely related to the cultivation of digitally capable technical talent (Ministry of Education of the People’s Republic of China, 2024). At the same time, compared with educational settings that place stronger emphasis on individual initiative, Chinese higher vocational classrooms often display more norm-oriented participation patterns (Ye & Nylander, 2024). This context provides a valuable setting for examining whether digital learning competence operates through differentiated participation mechanisms rather than through a single undifferentiated engagement process.

To address these gaps, this study draws on the 3P model to examine how digital learning competence is associated with learning performance among Chinese higher vocational college students. Specifically, it investigates the mediating roles of active learning participation and rule-based learning participation, as well as the moderating roles of teacher–student interaction and curriculum practicality. The study makes three contributions. First, it extends the 3P model to digital learning in higher vocational education by specifying digital learning competence as a presage factor, learning participation as the behavioral process, and learning performance as the product, while further treating teacher–student interaction and curriculum practicality as contextual conditions of the presage–process pathway. Second, it differentiates active participation from rule-based participation, thereby avoiding an overly broad treatment of learning participation and clarifying differentiated participation pathways. Third, it shows that digital learning competence is not automatically associated with learning performance; rather, this association may vary depending on whether supportive instructional contexts are linked to high-quality learning participation.

## 2. Theoretical Background and Hypotheses

### 2.1. The 3P Model of Learning

This study adopts Biggs’ presage–process–product (3P) model as its overarching theoretical framework. The model provides an analytical structure for explaining the relationships among presage factors, learning processes, and learning products (Biggs & Tang, 2007). In this study, digital learning competence is treated as a key presage variable, active learning participation and rule-based learning participation are treated as process variables, and learning performance is treated as the product variable. Accordingly, the proposed mediation structure follows the presage–process–product logic by examining whether digital learning competence is related to learning performance through two differentiated forms of learning participation. Because higher vocational education is strongly contextualized, teacher–student interaction and curriculum practicality are incorporated as contextual moderators of the association between the presage variable and the process variables. In this way, the moderation structure specifies the instructional conditions under which digital learning competence is more or less strongly associated with active or rule-based learning participation. This design is also supported by self-determination theory, which emphasizes the motivating role of social support (Ryan & Deci, 2000), and by experiential and situated learning theories, which highlight the importance of practical environments for learning and transfer (Kolb, 1984; Lave & Wenger, 1991). Given the cross-sectional data, this model should be understood as a theory-guided associational model rather than as a causal process model.

### 2.2. Digital Learning Competence and Learning Performance

In the digital era, digital learning competence has become an important factor associated with students’ learning performance. In this study, digital learning competence refers to learners’ basic qualities, including knowledge, skills, motivation, and attitudes, that enable them to use digital tools and digital resources for learning or to learn effectively in ubiquitous digital environments (Zhuang et al., 2018). This construct is related to, but distinct from, broader self-regulated learning abilities because it emphasizes learners’ capacity to use digital tools, resources, and environments to support learning. Learning performance refers to students’ academic outcomes after completing learning tasks and achieving expected knowledge and skill goals (E. Q. Xu, 2016). Previous studies have shown that digital technology, information literacy, and digital tool support are associated with or may support learning outcomes (T. Chen et al., 2020; G. H. Wang et al., 2021; Lei & Li, 2022). Research has also shown that digital learning competence is positively associated with learning engagement and learning outcomes (C. P. Chen et al., 2023; Hu et al., 2020; J. Zhang, 2025). Therefore, the following hypothesis is proposed.

**H1.**  *Digital learning competence is positively associated with students’ learning performance*.

### 2.3. The Mediating Role of Active Learning Participation

Learning participation refers to the time and effort students invest in academic activities. Prior studies have distinguished multiple forms of participation, including active and rule-based participation (Bao & Zhang, 2012; Liu et al., 2025). In this study, active learning participation refers to students’ voluntary inquiry, exploration, and self-initiated engagement beyond formal requirements. From the perspective of cognitive load theory, stronger digital learning competence may reduce the technical burden of digital learning and free cognitive resources for deeper learning, thereby increasing students’ willingness to engage actively (Sweller, 2011). Empirical evidence suggests that digital competence can promote academic engagement, especially in informal and self-directed learning contexts (Heidari et al., 2021). Active learning participation, in turn, is positively associated with academic performance and professional competence development (Uysal, 2025). Therefore, digital learning competence may be associated with learning performance through active learning participation. The following hypothesis is proposed:

**H2.**  *Active learning participation plays a mediating role in the association between digital learning competence and learning performance*.

### 2.4. The Mediating Role of Rule-Based Learning Participation

Rule-based learning participation refers to students’ academic behavioral performance in complying with basic institutional rules, classroom norms, and course-related requirements under external regulatory constraints (Bao & Zhang, 2012). Within the broader domain of behavioral engagement, it can be understood as the externally constrained and compliance-oriented component of students’ observable academic behavior. Compared with behavioral engagement as a broader construct, rule-based learning participation focuses more narrowly on whether students fulfill prescribed academic requirements in observable ways, such as attendance, procedural adherence, assignment submission, and completion of platform-based learning tasks. This compliance orientation refers to prescribed task completion rather than passive obedience or controlled participation. Digital learning competence may provide a technical foundation for such participation because students with stronger digital competence can complete attendance, submission, and platform-based learning tasks more efficiently (Romero-Rodríguez et al., 2020). In vocational settings, rule-based participation may also support basic academic achievement by maintaining task completion, procedural discipline, and minimum learning time (Broadbent & Poon, 2015). From this perspective, digital learning competence may be associated with learning performance through rule-based learning participation. The following hypothesis is proposed:

**H3.**  *Rule-based learning participation plays a mediating role in the association between digital learning competence and learning performance*.

### 2.5. The Moderating Role of Teacher–Student Interaction

Whether digital learning competence is associated with actual participation depends not only on students’ abilities but also on teacher–student interaction. Self-determination theory suggests that relatedness support is important for stimulating intrinsic motivation (Ryan & Deci, 2000). High-quality teacher–student interaction can provide emotional and instructional support, encouraging students with stronger digital competence to transform external tasks into active exploration (Chiu, 2021; Lang et al., 2022; Y. Yang, 2021). At the same time, stronger support may reduce students’ reliance on purely rule-based compliance and promote deeper meaning construction. Therefore, teacher–student interaction may strengthen the active pathway while weakening the rule-based pathway. The following hypotheses are proposed:

**H4a.**  *Teacher–student interaction positively moderates the relationship between digital learning competence and active learning participation*.

**H5a.**  *Teacher–student interaction negatively moderates the relationship between digital learning competence and rule-based learning participation*.

Building on these relationships, teacher–student interaction may further moderate the indirect effect of digital learning competence on learning performance through the two types of participation. In contexts characterized by high-quality teacher–student interaction, students with stronger digital learning competence may be more likely to transform their competence advantage into active exploration and high-quality learning participation, corresponding to a stronger indirect effect through active learning participation. By contrast, stronger teacher–student interaction may reduce dependence on mechanical rule compliance, corresponding to a weaker indirect effect through rule-based learning participation. In this study, “indirect effect” refers to the statistically estimated indirect pathway in the mediation model rather than to a strictly causal effect. The following hypotheses are proposed:

**H4b.**  *Teacher–student interaction positively moderates the indirect effect of digital learning competence on learning performance through active learning participation*.

**H5b.**  *Teacher–student interaction negatively moderates the indirect effect of digital learning competence on learning performance through rule-based learning participation*.

### 2.6. The Moderating Role of Curriculum Practicality

Experiential learning theory and situated learning theory suggest that deep learning is facilitated when abstract knowledge is embedded in authentic tasks and practical contexts (Kolb, 1984; Lave & Wenger, 1991). In curricula with high practicality, students with stronger digital learning competence may have more opportunities to apply digital resources to complex and meaningful tasks, and this may be associated with stronger active learning participation (Jing et al., 2025; M. Q. Yang, 2021). At the same time, highly practical learning contexts may shift students’ attention away from simple rule compliance and toward problem solving and application, thereby making the rule-based pathway less central. The following hypotheses are proposed:

**H6a.**  *Curriculum practicality positively moderates the relationship between digital learning competence and active learning participation*.

**H7a.**  *Curriculum practicality negatively moderates the relationship between digital learning competence and rule-based learning participation*.

Similarly, curriculum practicality may moderate the indirect associations between digital learning competence and learning performance through the two forms of participation. When curriculum practicality is high, students may be more likely to use digital competence for exploration, application, and problem solving, corresponding to a stronger indirect effect through active learning participation. Conversely, when authentic and practice-oriented tasks become more central, reliance on rule-based participation may become less important for the indirect effect through rule-based learning participation. The following hypotheses are proposed:

**H6b.**  *Curriculum practicality positively moderates the indirect effect of digital learning competence on learning performance through active learning participation*.

**H7b.**  *Curriculum practicality negatively moderates the indirect effect of digital learning competence on learning performance through rule-based learning participation*.

**Figure 1:**
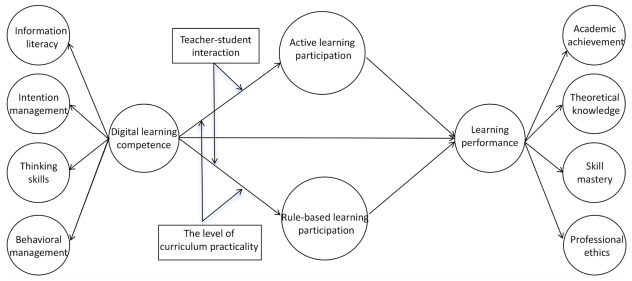
Proposed research model.

Based on the above theoretical analysis, this study proposes a dual-moderation, dual-mediation model, as shown in Figure 1.

## 3. Materials and Methods

### 3.1. Participants and Procedure

This study used a cross-sectional survey design. The data were drawn from the Vocational Education Student Development Survey conducted by the National Institute of Vocational Education at Beijing Normal University in December 2022. Using stratified proportional sampling, the survey covered 35 higher vocational colleges across seven provinces and municipalities in eastern, central, and western China. Within each institution, representative disciplinary clusters, including information technology, equipment manufacturing, medicine and health, and education and sports, were selected. Cluster sampling was then conducted at the class level by selecting two to three classes from Grades 1 to 3 within each major. The parent survey protocol, informed-consent materials, and recruitment materials were approved by the Research Ethics Review Committee of the Faculty of Education, Beijing Normal University (IRB No. BNU202210100036; approval date: 4 November 2022).

A total of 14,911 questionnaires were initially collected. Based on the research focus, the present study retained second- and third-year students from the three major categories with the highest participation frequency: equipment manufacturing, electronics and information, and medicine and health. This yielded 6182 cases meeting the requirements of major and grade level. The sample was further restricted to students with internship experience because internship-related learning contexts were central to the present analysis, resulting in 3752 eligible cases. After questionnaires with abnormal completion times (less than 5 min or more than 60 min) and cases with missing values on key variables were excluded, 3693 valid responses were retained for the final analysis, corresponding to a valid-case retention rate of 98.4% among eligible internship-experience cases.

### 3.2. Measures

Digital learning competence: Digital learning competence was measured using an assessment framework for vocational students’ digital learning competence, encompassing four dimensions: information literacy, intention management, thinking skills, and behavioral management (Tan et al., 2024; Zhuang et al., 2018). Although the intention-management and behavioral-management dimensions involve regulatory capacities, they were interpreted within the digital learning competence framework rather than as separate measures of general self-regulated learning. The scale included 29 items selected from the National Survey on Vocational Education Student Development and was rated on a 5-point Likert scale. Information literacy was measured with eight items, such as “I can proficiently use office software,” adapted from Bhandari et al. (2021). Intention management was measured with six items, such as “I usually have a way to achieve my goals,” adapted from the General Self-Efficacy Scale (C. K. Wang et al., 2001). Thinking skills were measured with 10 items, and behavioral management was measured with five items related to time, task, partner, and resource management. Confirmatory factor analysis showed acceptable construct validity (CFI = 0.95–0.97, TLI = 0.96–1.00, RMSEA < 0.09), and Cronbach’s alpha ranged from 0.82 to 0.94.

Learning performance: Learning performance was measured using four items adapted from the academic achievement dimension in Bao and Zhang’s (2012) survey on higher education quality and student development. One item captured students’ major ranking, coded as 5 for the top 25%, 3.75 for 26–50%, 2.5 for 51–75%, and 1.25 for the bottom 25%. The remaining three items were self-rated on a 5-point scale based on students’ perceived level of mastery. The final self-reported learning performance score was calculated as the mean of the four items, with higher scores indicating better perceived learning performance. Confirmatory factor analysis indicated good construct validity (CFI = 1.00, TLI = 1.00, RMSEA = 0.004), and Cronbach’s alpha was 0.79. This operationalization, although subjective, is consistent with prior work on higher vocational students’ learning performance (Lin, 2024).

Learning participation: Active learning participation and rule-based learning participation were specified as mediating variables. Both were measured using student participation items developed by Bao and Zhang (2012) and Guo and Wu (2017). The scale included 10 items on a 4-point Likert scale and was divided into two dimensions: active participation and rule-based participation. An example item for active learning participation was “actively participating in skills competitions.” The two subscales demonstrated acceptable construct validity (CFI = 0.91–1.00, TLI = 0.84–1.00, RMSEA < 0.08), and Cronbach’s alpha ranged from 0.72 to 0.85.

Teacher–student interaction and curriculum practicality: Teacher–student interaction and curriculum practicality were included as moderating variables. Both scales were adapted from Guo and Wu’s (2017) survey on talent cultivation and employment outcomes in local undergraduate institutions. Teacher–student interaction was measured with five items, such as “answering students’ questions after class,” using a 4-point Likert scale. Curriculum practicality was measured with four items related to curriculum design, such as “professional course instruction alternates with practical activities,” also using a 4-point Likert scale. The two subscales showed acceptable construct validity (CFI = 0.86–1.00, TLI = 0.84–1.00, RMSEA < 0.09), and Cronbach’s alpha ranged from 0.76 to 0.85.

### 3.3. Control Variables

Following previous research on digital learning competence and learning performance, this study included gender, grade level, major, and internet usage time as control variables (Li, 2021; Lin, 2024). All control variables were obtained from the student questionnaire.

### 3.4. Data Analysis

Data analysis was conducted in five steps. First, Harman’s single-factor test was performed to assess potential common method variance because all variables were collected through self-report questionnaires (Tang & Wen, 2020). Second, descriptive statistics and correlation analyses were conducted for all study variables. Third, confirmatory factor analysis and reliability analysis were used to examine the construct validity and internal consistency of the measurement scales. Fourth, SPSS 28.0 was used to conduct hierarchical regression analyses to estimate the direct associations and moderating effects of teacher–student interaction and curriculum practicality. Continuous predictors involved in interaction terms were mean-centered before the interaction terms were created, and simple slope analyses were conducted for significant interaction terms. Fifth, the PROCESS 4.0 macro was used to estimate indirect effects and moderated mediation effects through bootstrap estimation, following procedures for mediation analysis recommended by Wen and Ye (2014). Because of the cross-sectional design, these effects should be interpreted as statistically estimated effects within the specified models rather than as causal effects.

## 4. Results

### 4.1. Common Method Variance

Because the data were collected through self-report questionnaires, Harman’s single-factor test was conducted to assess potential common method variance, following the recommendation of Tang and Wen (2020). Seven factors with eigenvalues greater than 1 were extracted, and the first factor accounted for 41.27% of the total variance, which was below the commonly used 50% threshold. This result suggests that common method variance was unlikely to dominate the observed relationships, although it cannot fully rule out all forms of self-report bias.

### 4.2. Descriptive Statistics and Correlations

Table 1 presents the means, standard deviations, and correlations of the main variables. Digital learning competence was positively correlated with learning performance (r = 0.464, *p* < 0.01), active learning participation (r = 0.187, *p* < 0.01), teacher–student interaction (r = 0.528, *p* < 0.01), and curriculum practicality (r = 0.675, *p* < 0.01), and negatively correlated with rule-based learning participation (r = −0.034, *p* < 0.05). Teacher–student interaction and curriculum practicality were also positively associated with learning performance. These findings provided preliminary support for the subsequent model testing.

### 4.3. Direct Associations and Indirect Effects

SPSS 28.0 was used for regression analyses, and PROCESS 4.0 was employed to estimate mediation, moderation, and moderated mediation models. Unstandardized PROCESS estimates are reported in Table 2, Standardized regression coefficients are reported in Table 3, and further unstandardized PROCESS estimates are reported in Table 4.

After controlling for gender, major, grade level, and internet usage time, digital learning competence was positively associated with learning performance (β = 0.460, *p* < 0.001), supporting H1. Digital learning competence was also positively associated with active learning participation (β = 0.176, *p* < 0.001), and active learning participation was positively associated with learning performance (β = 0.039, *p* < 0.05). Bootstrap analysis further showed that the indirect effect through active learning participation was significant but small in magnitude (effect = 0.010, 95% CI [0.003, 0.020]), supporting H2.

By contrast, digital learning competence was negatively associated with rule-based learning participation (β = −0.031, *p* < 0.05), but the indirect effect through rule-based learning participation in the relationship between digital learning competence and learning performance was not significant (effect = −0.005, 95% CI [−0.012, 0.001]). Therefore, H3 was not supported. These findings suggest that digital learning competence was more consistently associated with learning performance through active learning participation than through rule-based learning participation.

### 4.4. Moderating Effects

**Figure 2:**
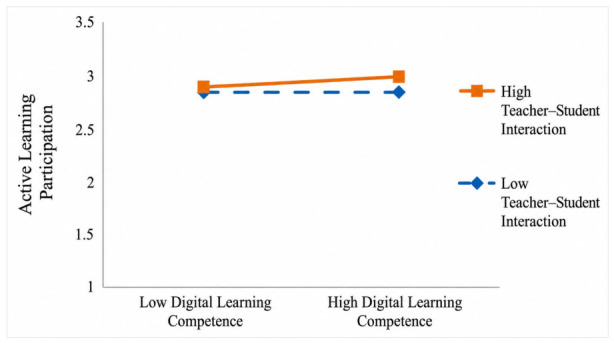
Moderating role of teacher–student interaction in the relationship between digital learning competence and active learning participation.

To test the moderating role of teacher–student interaction, all relevant variables were mean-centered and interaction terms were created. As shown in Model 8 of Table 3, the interaction between digital learning competence and teacher–student interaction was positively associated with active learning participation (β = 0.017, *p* < 0.05). Simple slope analysis indicated that the positive relationship between digital learning competence and active learning participation was stronger at higher levels of teacher–student interaction and weaker at lower levels. As shown in Figure 2, the positive association between digital learning competence and active learning participation was stronger under conditions of high teacher–student interaction and relatively weaker under conditions of low teacher–student interaction. Thus, H4a was supported, although the moderation effect was modest in magnitude.

**Figure 3:**
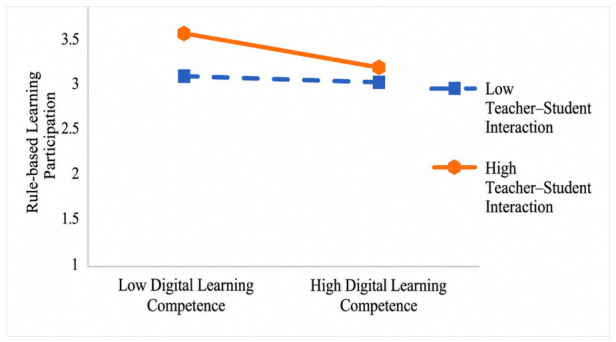
Moderating role of teacher–student interaction in the relationship between digital learning competence and rule-based learning participation.

As shown in Model 12 of Table 3, the interaction between digital learning competence and teacher–student interaction was negatively associated with rule-based learning participation (β = −0.079, *p* < 0.01). Further simple slope analysis showed that the negative relationship between digital learning competence and rule-based learning participation was stronger at higher levels of teacher–student interaction and weaker at lower levels. As shown in Figure 3, the negative association between digital learning competence and rule-based learning participation was stronger under conditions of high teacher–student interaction and relatively weaker under conditions of low teacher–student interaction. Thus, H5a was supported, but the effect should be interpreted cautiously.

**Figure 4:**
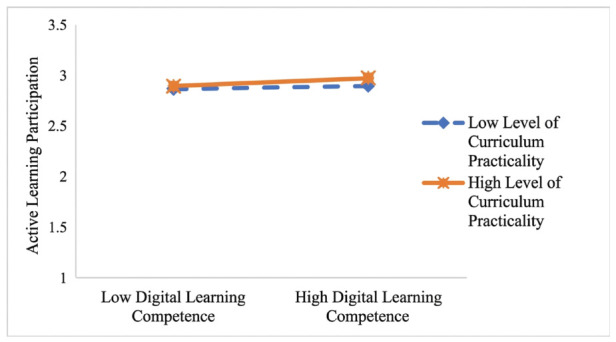
Moderating role of curriculum practicality in the relationship between digital learning competence and active learning participation.

To test the moderating role of curriculum practicality, variables were similarly mean-centered and interaction terms were entered into the hierarchical regression model. Model 9 of Table 3 shows that the interaction between digital learning competence and curriculum practicality was positively associated with active learning participation (β = 0.011, *p* < 0.05). Simple slope analysis further indicated that the positive association between digital learning competence and active learning participation was stronger at higher levels of curriculum practicality and weaker at lower levels. As shown in Figure 4, this positive association was stronger when curriculum practicality was high and weaker when curriculum practicality was low. Thus, H6a was supported, although the incremental explanatory contribution was small.

**Figure 5:**
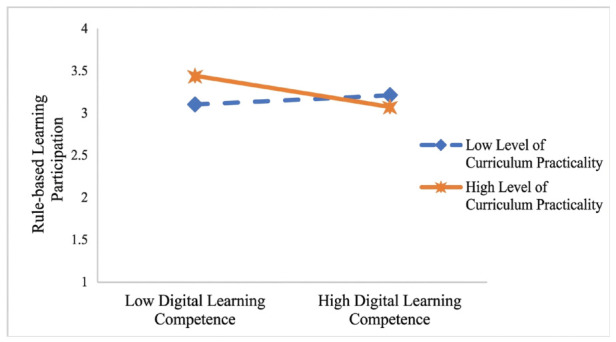
Moderating role of curriculum practicality in the relationship between digital learning competence and rule-based learning participation.

As shown in Model 13 of Table 3, the interaction between digital learning competence and curriculum practicality was negatively associated with rule-based learning participation (β = −0.119, *p* < 0.001). Simple slope analysis indicated that the negative association between digital learning competence and rule-based learning participation was stronger at higher levels of curriculum practicality and weaker at lower levels. As shown in Figure 5, this negative association was stronger when curriculum practicality was high and relatively weaker when curriculum practicality was low. Thus, H7a was supported, with a small incremental effect.

The regression results for the main, mediating, and moderating analyses are summarized in Table 3.

### 4.5. Moderated Mediation Analyses

Building on the above moderating effects, the bootstrap method was used to estimate moderated mediation effects. The results are shown in Table 4. In the active learning participation pathway, both teacher–student interaction and curriculum practicality positively moderated the indirect effect. Specifically, the differences in the indirect effects between the high and low teacher–student interaction groups and between the high and low curriculum practicality groups were significant. The indirect effects were stronger under conditions of high teacher–student interaction and high curriculum practicality than under the corresponding low-level conditions, indicating that a more positive teacher–student interaction environment and higher curriculum practicality were associated with a stronger indirect pathway through active learning participation. Therefore, H4b and H6b were supported.

In the rule-based learning participation pathway, teacher–student interaction and curriculum practicality showed significant conditional effects, but the findings were not fully consistent with the original hypotheses. Under the moderation of teacher–student interaction, the difference in indirect effects between the high and low groups was significant; however, only the low teacher–student interaction condition produced a zero-excluding confidence interval. Therefore, H5b received partial support. Under the moderation of curriculum practicality, the indirect effect was significant in the high curriculum-practicality condition but not in the low condition, indicating that curriculum practicality changed the conditions under which rule-based learning participation functioned as a mediator. Because this pattern did not show the expected weakening of the indirect effect, H7b was not supported. Importantly, these conditional rule-based effects should be interpreted in relation to the positive total and direct associations between digital learning competence and learning performance.

## 5. Discussion

### 5.1. Active Learning Participation as the Primary Pathway to Learning Performance

This study found that digital learning competence was positively associated with higher vocational students’ self-reported learning performance and also showed a small positive indirect effect through active learning participation. This finding is broadly consistent with previous research showing that digital competence is associated with learning engagement and learning outcomes (Dang et al., 2024; Zhao et al., 2021), and with evidence from Chinese higher vocational education indicating that digital technology is related to student outcomes through learning experience and learning engagement (X. Zhang et al., 2024).

More importantly, this study shows that active learning participation is not simply a general form of participation but the most consistent process pathway through which digital learning competence is associated with learning performance. Recent studies have emphasized the multidimensional nature of student participation and suggested that different forms of participation do not necessarily serve the same function (Bergdahl et al., 2024; Heilporn et al., 2024). Building on this line of work, the present findings indicate that, in higher vocational digital learning contexts, what matters most is not participation in a broad sense but whether students engage actively in exploration, reflection, and knowledge construction.

This result is closely related to the practice-oriented nature of higher vocational education. Compared with general academic learning, higher vocational learning places greater emphasis on problem solving and practical operation. In this context, the value of digital learning competence lies not merely in helping students complete assigned tasks more efficiently but in supporting students’ regulation of learning processes and use of digital tools for practical problem solving (Deschênes et al., 2024). From this perspective, extending the 3P model to higher vocational digital learning helps clarify that the educational value of digital learning competence lies not in a simple instrumental gain but in whether it is accompanied by higher-quality learning participation and performance.

### 5.2. Rule-Based Learning Participation as a Conditional Externally Regulated Pathway

The interpretation of rule-based learning participation in this section should not be understood as equating this construct with passive obedience or simple compliance. Rather, the following discussion uses its externally regulated and prescribed-task-oriented features to highlight the distinctive nature of this form of academic participation in vocational education contexts.

In contrast to the active learning participation pathway, rule-based learning participation did not emerge as a stable positive mediating pathway. This finding is not fully consistent with studies that treat learning participation as a generally positive variable, but it is consistent with recent arguments that participation is multidimensional and functionally differentiated (Bergdahl et al., 2024; Heilporn et al., 2024). In particular, behavioral participation tends to be reflected in attendance, task completion, and procedural adherence, whereas deeper forms of engagement are more closely associated with self-regulation, reflection, and meaning construction. The present findings further suggest that rule-based learning participation should not be regarded as a pathway parallel to, and equally stable as, active learning participation.

This pattern may be explained by the characteristics of both higher vocational education and digital learning environments. Higher vocational education emphasizes standardized operation, procedural execution, and task completion, which makes rule-based participation highly visible and practically necessary. At the same time, digital platforms often convert learning behavior into recordable actions such as checking in, submitting assignments, and completing prescribed steps. Such indicators reflect behavioral or task-completion-oriented participation, but they do not necessarily indicate high-quality cognitive engagement. In this sense, completing tasks does not necessarily mean that students are genuinely engaged in learning.

This interpretation is supported by studies showing that classroom or online interaction may be associated with students’ engagement and self-directed learning, but such associations often operate through emotional and behavioral involvement rather than directly indicating deep understanding (Gherghel et al., 2023). Reviews of authentic assessment and task design further indicate that critical thinking, problem solving, and collaboration are more likely to be fostered by high-quality tasks embedded in authentic contexts than by procedural completion alone (Vlachopoulos & Makri, 2024). Accordingly, in higher vocational digital learning, rule-based learning participation is understood as a conditional externally regulated pathway. It helps maintain basic learning order, but it is not as consistently associated with learning performance as active learning participation. The negative conditional indirect effects should not be read as evidence that digital learning competence reduces learning performance overall; rather, they indicate that students with stronger digital competence may rely less on externally regulated and prescribed-task-oriented participation under some instructional conditions, even while the overall association between digital competence and performance remains positive. Distinguishing between active and rule-based participation may therefore offer a more nuanced explanation of digital learning processes in higher vocational education than treating participation as a single construct.

### 5.3. The Moderating Roles of Teacher–Student Interaction and Curriculum Practicality

This study further found that teacher–student interaction and curriculum practicality were not associated with all forms of participation in the same way. Rather, they were associated with a stronger pathway linking digital learning competence to active learning participation. This finding is broadly consistent with previous research suggesting that teacher support, teacher–student interaction, and authentic tasks are associated with stronger student engagement (Sun et al., 2022; X. Xu et al., 2023). It should also be noted that several moderation estimates were statistically significant but small in magnitude, especially given the large sample size. Therefore, these findings should be interpreted as modest conditional associations rather than as evidence of substantial educational effects.

Compared with studies that treat instructional context as a generally facilitating factor, the present study further suggests that the role of teacher–student interaction and curriculum practicality is not simply to increase students’ overall participation but to activate active learning participation more specifically. In other words, these contextual variables appear to be related not only to whether students participate but also to how they participate. This interpretation is consistent with findings from Chinese higher vocational education showing that the effects of digital technology on student outcomes often operate through process variables such as learning experience and learning engagement rather than through direct linear effects (X. Zhang et al., 2024).

By contrast, the patterns involving teacher–student interaction and curriculum practicality in the rule-based learning participation pathway were more complex and did not show the same stable positive pattern. This indicates that instructional context does not relate to the educational value of all forms of participation in the same way but is more likely to benefit forms associated with active exploration and knowledge construction. In courses with high practicality, authentic tasks often require judgment, transfer, and problem solving rather than merely completing prescribed steps (Luk & Chan, 2024; Vlachopoulos & Makri, 2024). Thus, digital learning competence is not automatically associated with learning performance; this association may be stronger in instructional contexts characterized by teacher–student interaction and practice-oriented curricula. From this perspective, instructional context should not be treated as a neutral background condition in the 3P model but as an important condition shaping whether digital learning competence can be linked to high-quality learning participation.

### 5.4. Practical Implications

The findings suggest that although higher vocational colleges have made progress in digital teaching reform, digital learning competence is not automatically associated with higher learning performance. Its educational value may be more likely to appear when it is accompanied by active learning participation and supportive instructional contexts. Three practical implications follow. First, the cultivation of digital learning competence should be integrated with active, inquiry-based learning tasks so that students move from being able to use technology to using technology for exploration, problem solving, and reflection. Second, digital learning evaluation should not equate task completion, platform records, or attendance with high-quality learning; instead, it should include evidence of application, collaboration, and problem solving. Third, teachers should use timely feedback, dialogic interaction, and practice-oriented curriculum design to support students in using digital competence for meaningful learning behaviors.

### 5.5. Limitations and Future Research

Several limitations should be acknowledged. First, this study relied mainly on students’ self-reported data. Although anonymity was ensured and Harman’s single-factor test was used as a diagnostic check, self-report bias may still have affected the findings. Thus, the results mainly reflect students’ subjective perceptions. The cross-sectional design also means that the mediation and moderation results should be interpreted as associational rather than causal. Second, although the sample covered colleges in eastern, central, and western China, remote-area institutions and students without internship experience were underrepresented, which may limit generalizability. Third, the measure of digital learning competence captured general perceived competence but did not assess among specific platforms, technologies, AI-based tools, AI literacy, or teacher-side digital competencies.

Future research could use multiple data sources, such as platform logs, classroom observations, teacher surveys, teacher evaluations, objective academic records, and learning analytics. It could also broaden the sampling scope and adopt longitudinal or experimental designs. Future studies could further examine learning motivation, teacher feedback quality, students’ AI literacy and generative AI use, AI-supported learning practices, teachers’ digital and pedagogical competencies, and the specific technologies used in vocational courses.

## 6. Conclusions

Using the 3P framework and a learning participation perspective, this study examined how digital learning competence is associated with learning performance among Chinese higher vocational students. The results indicate that digital learning competence was positively associated with learning performance and showed a small indirect association with learning performance through active learning participation. In contrast, rule-based learning participation did not function as a stable positive mediator and appeared to be more context-dependent. Teacher–student interaction and curriculum practicality further moderated this process and were associated with a stronger pathway from digital learning competence to active learning participation. Supportive teacher–student interaction and higher curriculum practicality were associated with stronger links between digital learning competence and active exploration, reflection, and knowledge construction, which were, in turn, related to learning performance. These findings suggest that the educational value of digital learning competence in higher vocational education lies less in helping students complete tasks more efficiently than in enabling more active and deeper learning within supportive instructional contexts. Because the data were cross-sectional and self-reported, these conclusions should be interpreted as theoretically informed associations rather than causal evidence.

## Figures and Tables

**Table 1:** Descriptive statistics and correlations of the main variables.

Variables	Mean	SD	1	2	3	4	5	6
1. Digital learning competence	3.362	0.683	1					
2. Active learning participation	2.953	0.769	0.187 **	1				
3. Rule-based learning participation	3.197	0.863	−0.034 *	−0.035 *	1			
4. Learning performance	3.436	0.910	0.464 **	0.129 **	0.106 **	1		
5. Teacher–student interaction	3.196	0.962	0.528 **	0.176 **	0.079 **	0.316 **	1	
6. Curriculum practicality	3.236	0.619	0.675 **	0.178 **	0.007	0.385 **	0.653 **	1

Notes: N = 3693. * *p* < 0.05; ** *p* < 0.01.

**Table 2:** Bootstrap results for the mediation analyses.

Effect Type	Effect Size	Standard Error	Bootstrap CI Lower Bound	Bootstrap CI Upper Bound
Total effect	0.608	0.019	0.571	0.646
Direct effect	0.603	0.019	0.565	0.641
Active pathway: digital learning competence → active learning participation → learning performance	0.010	0.004	0.003	0.020
Rule-based pathway: digital learning competence → rule-based learning participation → learning performance	−0.005	0.003	−0.012	0.001

Notes: Bootstrap confidence intervals are reported at the 95% level. The entries are unstandardized PROCESS estimates.

**Table 3:** Regression results for the main, mediating, and moderating analyses of digital learning competence and learning performance.

(**A**) Learning performance as the outcome variable					
**Variables**	**M1**	**M2**	**M3**	**M4**	**M5**
Gender	0.003	0.020	0.023	0.009	0.011
Major	0.016	0.049	0.050 ***	0.047	0.048
Grade level	0.132 ***	0.104 ***	0.101 ***	0.103 ***	0.100 ***
Internet usage time	−0.075 ***	−0.044	−0.044	−0.039	−0.038
Digital learning competence		0.460 ***	0.453 ***	0.464 ***	0.456 ***
Active learning participation			0.039 *		0.042
Rule-based learning participation				0.116 ***	0.117 ***
R^2^	0.024	0.232	0.234	0.246	0.247
ΔR^2^	0.024	0.208	0.001	0.013	0.015
ΔF	22.986 ***	999.781 ***	6.945	64.618 ***	36.426 ***
(**B**) Active learning participation as the outcome variable					
**Variables**		**M6**	**M7**	**M8**	**M9**
Gender		−0.075 ***	−0.069 ***	−0.027 ***	−0.026 ***
Major		−0.036	−0.024	−0.008	−0.006
Grade level		0.085 ***	0.075	0.025 ***	0.024 ***
Internet usage time		−0.025	−0.013 ***	−0.002	−0.002
Digital learning competence			0.176 ***	0.026 ***	0.027 ***
Teacher–student interaction				0.034 **	
DLC × teacher–student interaction				0.017 *	
Curriculum practicality					0.026 *
DLC × curriculum practicality					0.011 *
R^2^		0.016	0.046	0.049	0.052
ΔR^2^		0.016	0.031	0.003	0.001
ΔF		14.689 ***	118.039 ***	10.295 *	4.112
(**C**) Rule-based learning participation as the outcome variable					
**Variables**		**M10**	**M11**	**M12**	**M13**
Gender		0.098 ***	0.097 ***	0.172 ***	0.173 ***
Major		0.018 ***	0.016	0.008	0.022
Grade level		0.004	0.006	0.006	0.007
Internet usage time		−0.048 *	−0.050 *	−0.042 *	−0.043 *
Digital learning competence			−0.031 *	−0.112	−0.065 *
Teacher–student interaction				0.159 **	
DLC × teacher–student interaction				−0.079 **	
Curriculum practicality					0.052
DLC × curriculum practicality					−0.119 ***
R^2^		0.013	0.014	0.027	0.021
ΔR^2^		0.013	0.001	0.002	0.006
ΔF		12.075 ***	3.631	9.105 *	20.592 ***

Notes: DLC = digital learning competence. Coefficients are standardized regression coefficients. Control variables were included in all models. The ΔF row reports the change statistic for the newly added predictor or predictor block. * *p* < 0.05; ** *p* < 0.01; *** *p* < 0.001.

**Table 4:** Results of the moderated mediation analyses.

Moderator Level	Active Effect	SE	95% CI	Rule-Based Effect	SE	95% CI
High teacher–student interaction (M + 1 SD)	0.008	0.003	[0.002, 0.015]	−0.020	0.005	[−0.030, 0.010]
Low teacher–student interaction (M − 1 SD)	0.003	0.002	[0.0004, 0.0077]	−0.008	0.005	[−0.0172, −0.0006]
Difference between high and low groups	0.004	0.002	[0.0007, 0.0080]	−0.010	0.004	[−0.019, −0.002]
High curriculum practicality (M + 1 SD)	0.007	0.003	[0.002, 0.014]	−0.017	0.005	[−0.027, −0.008]
Low curriculum practicality (M − 1 SD)	0.005	0.002	[0.0009, 0.0094]	0.001	0.004	[−0.0072, 0.0092]
Difference between high and low groups	0.002	0.002	[0.0001, 0.0061]	−0.015	0.004	[−0.024, −0.007]

Notes: Active effect refers to the indirect effect through active learning participation; rule-based effect refers to the indirect effect through rule-based learning participation. CI = confidence interval. Effects are unstandardized PROCESS estimates; CIs excluding zero indicate statistical significance.

## Data Availability

To protect participant confidentiality, the dataset is not publicly posted. De-identified data supporting the findings of this study are available from the corresponding author upon reasonable request, subject to institutional and ethical restrictions.
